# Prioritizing Patients from the Most Deprived Areas on Elective Waiting Lists in the NHS in England: Estimating the Health and Health Inequality Impact

**DOI:** 10.1177/23814683241310146

**Published:** 2025-01-21

**Authors:** Naomi Kate Gibbs, Susan Griffin, Nils Gutacker, Adrián Villaseñor, Simon Walker

**Affiliations:** Centre for Health Economics, University of York, Heslington, York, UK; Centre for Health Economics, University of York, Heslington, York, UK; Centre for Health Economics, University of York, Heslington, York, UK; Centre for Health Economics, University of York, Heslington, York, UK; Centre for Health Economics, University of York, Heslington, York, UK

**Keywords:** health economics, decision modelling, health policy, health inequality, elective surgery

## Abstract

**Introduction.** Reducing hospital waiting lists for elective procedures is a policy concern in the National Health Service (NHS) in England. Following growth in waiting lists after COVID-19, the NHS published an elective recovery plan that includes an aim to prioritize patients from deprived areas. We use a previously developed model to estimate the health and health inequality impact under hypothetical targeted versus universal policies to reduce waiting time. **Methods.** We use a Markov model to estimate the health impact of waiting, by index of multiple deprivation quintile group, for 8 elective procedures. We estimate patients’ remaining quality-adjusted life-years (QALYs) with baseline waiting times and under 2 hypothetical policy scenarios: 1) a universal policy in which all patients receive an equal reduction in wait and 2) a targeted policy in which patients living in the most deprived quintile are prioritized. We estimate individual and population level health under each of the 2 policies and compare it with baseline. We also estimate how health inequality changes from baseline using the slope index of inequality, reflecting the difference in health between the least and most deprived quintile based on QALYs. **Results.** A universal reduction in waiting time is estimated to improve overall population health but increase health inequality. A targeted reduction would achieve nearly the same overall health gain and would also increase population-level health inequalities but to a lesser extent than the universal policy would. **Discussion.** If the NHS is successful in prioritizing patients on waiting lists from the most deprived areas, this may result in smaller increases in health inequalities while maintaining a similar level of overall health gain compared with a universal policy.

**Highlights:**

- The NHS elective recovery plans include prioritizing patients who live in the most deprived areas of England.
- Evaluating a hypothetical targeted wait time reduction policy against a universal wait time reduction policy suggests almost the same level of population health gain could be achieved while lessening the negative impact on health inequality.
- Expected outcomes of government health policies should be quantified to explore the impact on both health maximization and health inequality minimization, as both represent legitimate policy concerns.

## Introduction

In the National Health Service (NHS) in England (a publicly funded health care system), there are historically high numbers of patients waiting for nonemergency (i.e., elective) hospital treatment. These long waits result in lifetime health losses to the patient that vary by procedure and by patient group.^1^ In England, waiting list numbers are regularly published and considered an indication of the state of the health service.^2,3^ The NHS constitution includes a universal target that 92% of patients on inpatient waiting lists for elective procedures should be treated within 18 wk, which was last met in 2015.^4,5^ COVID-19 resulted in a sharp increase in numbers on the waiting lists, raising the topic up the policy agenda.^6^ This was coupled with an increasing awareness, and policy concern, for population-level health inequalities brought into focus by higher death rates in global majority groups and those living in more deprived areas in England during the pandemic.^7–9^ Consequently, elective recovery plans published by the NHS stated that priority should be given to the most deprived index of multiple deprivation (IMD) quintile group and Black and minority ethnic populations on the waiting list.^10^

Prioritizing one group over another will not only redistribute health care, and therefore health outcomes, in a population but may also affect the overall level of health a health care system can achieve. Policy makers regularly face decisions in which they are trading off health maximization and health inequality minimization, although this is rarely explicit. Methods exist to quantify health inequality policy effects, but this is not currently part of routine practice, with decisions for new health technologies in the NHS in England based on health maximization principles.^11,12^ The concern for health inequalities found in policy documents suggests that analysis that provides information of the policy impact on health inequalities is important and should become part of standard reporting for research studies to inform decision making. The general public in England appears to agree that this should be a policy concern, with research suggesting they value health gains to the poorest quintile more than health gains to the richest quintile of the population.^13^

We use our previously published waiting times model to consider 2 hypothetical policy scenarios for addressing waiting times: 1) a universal policy that reduces waiting equally for all IMD quintile groups and 2) a targeted policy that allocates more of the reduction in waiting to the most deprived IMD quintile group, while keeping the total reduction in wait equal to scenario 1. The model estimates the health outcomes, measured in quality-adjusted life-years (QALYs), of waiting across 8 high-volume elective procedures, in the NHS in England.^14^ Our model estimated differential mortality and morbidity impacts by quintile group, with more deprived groups benefiting less from a reduction in waiting time due to their lower life expectancy and health-related quality of life. We also found from English hospital data a relatively larger share of less deprived groups among the patient population (i.e., less deprived individuals are more likely to undergo elective procedures). To explore how these factors might affect health and equality outcomes when faced with a policy change, we model the 2 hypothetical policy scenarios described above. Both policies lead to the same total reduction in weeks of waiting across all patients for a particular procedure. The QALY estimates under each policy, by IMD quintile group, are used to estimate total health gain and change in population-level health inequality compared with baseline waiting times. Using our modeling framework applied to each policy, we aim to demonstrate how government can consider the tradeoffs between different prioritization strategies.

## Methods

### Overview

Using a previously developed waiting times model applied to 8 elective procedures (cataract, coronary artery bypass graft [CABG], cholecystectomy, hernia, hip replacement, hysterectomy, knee replacement, and percutaneous coronary intervention [PCI]),^1,14^ we estimate the remaining QALYs for a typical patient (mean age, comorbidities, and waiting time) in each IMD quintile group for 2 hypothetical waiting-time reduction policies and compare them with outcomes under baseline waiting times. The first policy is a universal reduction in waiting times across all IMD quintile groups and the second is targeted, giving a larger share of the overall reduction in wait to IMD quintile group 1 (the most deprived quintile group). Both policies lead to the same total reduction in weeks of waiting across all patients for a particular procedure. We explore the population-level health gain by multiplying these individual impacts by the estimated patient population. We estimate the health inequality impact by calculating the change in the slope index of inequality (SII) for each scenario compared with baseline.

### Model

**Figure 1:**
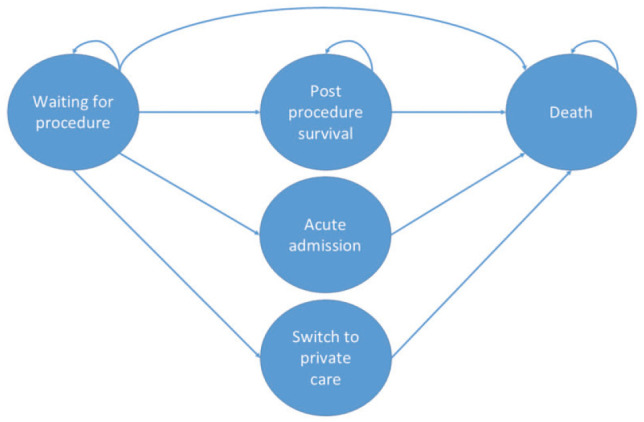
Simplified model schematic.

Our waiting times model was purposely built to accommodate a range of procedures using routine data wherever possible. In brief, it consists of a Markov model capturing health pre- and postprocedure from the time of entering the waiting list. It includes the possibility of exiting preprocedure to nonelective NHS care, self-funded private care, or death (Figure 1). The model runs in weekly cycles to estimate the remaining discounted lifetime QALYs (from the point at which a patient is added to the waiting list) for 10 subgroups defined by sex and IMD quintile groups^15^ for each procedure. Inputs are stratified by IMD quintile group specifically to capture the inequality impact, which includes pre- and postprocedure survival, health-related quality-of-life scores, probability of exiting to acute procedure, and probability of exiting to private care. Outcomes are discounted at 3.5% in line with UK guidance.^16^ The model was populated with data from routinely collected datasets (Hospital Episode Statistics [HES], Patient-Reported Outcome Measures, and Office for National Statistics Mortality records), supplemented by the academic literature. Full details are available.^1,14^

### Patient Population and Baseline Wait

The routine data available are limited to those who received the procedure as opposed to all those who entered the waiting list.^17^ Therefore, we estimate a cohort of patients who enter the inpatient wait list using the observed number of patients receiving procedures on the NHS in 2019–2020 using HES data, increased to account for those who die, leave for private care, or acute admission (all IMD quintile specific) as predicted by our model given observed waiting times (Table 1).^1^ Further baseline characteristics of HES patients across these procedures have been previously published.^1^

**Table 1:** Estimated Numbers Entering the Waiting List (and Percentage Share) Based on 2019–2020 HES Data and Our Model Projections

	Q1 (Most Deprived)	Q2	Q3	Q4	Q5
CABG	1,123 (16%)	1,311 (19%)	1,461 (21%)	1,648 (23%)	1,542 (22%)
Cataract	50,903 (16%)	56,010 (17%)	65,075 (20%)	74,492 (23%)	78,013 (24%)
Cholecystectomy	28,201 (19%)	28,884 (20%)	30,077 (20%)	30,157 (20%)	29,840 (20%)
Hernia	9,716 (15%)	11,387 (17%)	13,102 (20%)	15,095 (23%)	16,154 (25%)
Hip replacement	8,226 (11%)	11,644 (16%)	15,050 (21%)	17,910 (25%)	19,596 (27%)
Hysterectomy	7,245 (18%)	7,753 (19%)	8,150 (20%)	8,496 (21%)	8,395 (21%)
Knee replacement	10,274 (13%)	13,537 (17%)	16,751 (21%)	19,255 (24%)	19,492 (25%)
PCI	3,353 (16%)	3,850 (18%)	4,344 (20%)	4,748 (22%)	5,168 (24%)

CABG, coronary artery bypass graft; HES, Hospital Episode Statistics; PCI, percutaneous coronary intervention.

These estimates are taken from table 5 in reference 1.

The baseline mean waiting time, from referral to treatment, for each quintile for each procedure is estimated using HES data for 2019–2020. Outliers were removed using plus/minus 3 times the standard deviation (Table 2). For most procedures, more deprived groups are waiting longer than the least deprived.

**Table 2:** Baseline Mean Weeks Wait per Patient, Using the 2019–2020 HES Sample

	Q1 (Poorest)	Q2	Q3	Q4	Q5
CABG	9.6	9.1	9.5	8.9	8.2
Cataract	10.4	11.2	11.7	11.4	11.4
Cholecystectomy	13.0	12.3	12.0	11.5	10.9
Hernia	12.5	12.3	12.4	11.9	11.5
Hip replacement	15.9	15.8	15.8	15.2	14.3
Hysterectomy	15.0	15.9	15.5	15.0	14.5
Knee replacement	16.4	16.7	16.7	16.1	15.3
PCI	8.5	8.4	8.1	8.2	7.5

CABG, coronary artery bypass graft; HES, Hospital Episode Statistics; PCI, percutaneous coronary intervention.

### Policy Scenarios

We estimate outcomes under 2 scenarios and compare them to baseline waiting times.

1. A universal reduction of a 4-wk wait for all patients across all IMD quintile groups
2. A reduction of a 6-wk wait for patients in IMD quintile group 1 (the most deprived); an equal wait reduction across all other IMD groups is then determined based on the total reduction in waiting across all patients equaling that from the 4-wk scenario (Table 3)

**Table 3:** Number of Weeks’ Reduction in Wait, per Patient, by Procedure for Each Scenario

	Universal Weeks Wait Reduction	Targeted Weeks Wait Reduction	
	Q1–Q5	Q1 (Poorest)	Q2–Q5
CABG	4	6	3.6
Cataract	4	6	3.6
Cholecystectomy	4	6	3.5
Hernia	4	6	3.7
Hip replacement	4	6	3.7
Hysterectomy	4	6	3.6
Knee replacement	4	6	3.7
PCI	4	6	3.6

CABG, coronary artery bypass graft; PCI, percutaneous coronary intervention.

The targeted policy (2) effectively redistributes the total reduction in weeks’ wait available for that patient population to favor the most deprived quintile. This assumes reducing waiting by 1 wk for patients in quintile 1 is perfectly exchangeable, from a resourcing perspective, to reducing waiting by 1 wk for a patient in any other quintile. This relates only to exchanging waiting time within, and not between, procedures.

### Analysis

#### Population health impact

We run the model for each procedure for a typical (mean age, comorbidities, and waiting time) individual in each quintile group at baseline for each scenario. We then estimate the increase in QALYs, by procedure and IMD quintile group, compared with baseline (Table 2).

We multiply the mean individual increase in QALYs compared with the baseline (by procedure and IMD quintile group) by the numbers entering the waiting list (Table 1) to calculate population health gain for the reduced wait. The aggregation of this across IMD quintile groups is the total population health gain by procedure. This is completed for both scenarios and for baseline. We also aggregate across all 8 procedures to estimate the total impact under each scenario and baseline.

#### Health inequality impact

We estimate the impact of the 2 policies on population health inequality using the SII.^18^ This is a simple regression method intuitively interpreted as the difference in health (in this study measured as population average QALYs) between the most and least deprived quintile. A high SII would indicate a greater difference in health between the most and least deprived; if positive, the least deprived have the best health, and if negative, the more deprived groups have the best health. A low SII would indicate little difference in health between the most and least deprived.

For each policy scenario, we estimate the total increase in remaining lifetime QALYs for each IMD quintile across the patient population undergoing the procedure. This patient population QALY increase is then added to the total lifetime QALYs by IMD for the whole population, and the postpolicy lifetime QALYs for the whole population by IMD quintile from each scenario are estimated. The change in the SII compared with baseline indicates the impact on health inequality at a population level. We estimate this for each procedure and aggregating across all 8 procedures. This methodology is based on distributional cost-effectiveness analysis.^12,19^

The baseline quality-adjusted life expectancy and populations are taken from Love-Koh et al.^20^ and the Office for National Statistics for 2019–2020, respectively (Table 4).

**Table 4:** Estimated Population and Quality-Adjusted Life Expectancy in England

Quintile	General Population from April 2019 to March 2020 (Office for National Statistics, 2023)	Baseline Individual Quality-Adjusted Life Expectancy (Love-Koh et al., 2023^20^)
Q1 (poorest)	11,303,662	62.2
Q2	11,617,815	65.5
Q3	11,467,307	69.5
Q4	11,158,269	71.1
Q5	10,940,630	73.3

#### Sensitivity analysis

We estimate the results with an equal share of patients across the 5 quintiles while maintaining the total procedure-specific patient population (Appendix Table 1). This enables us to explore the extent to which the results are driven by the share of patients across the quintiles.

## Results

**Figure 2:**
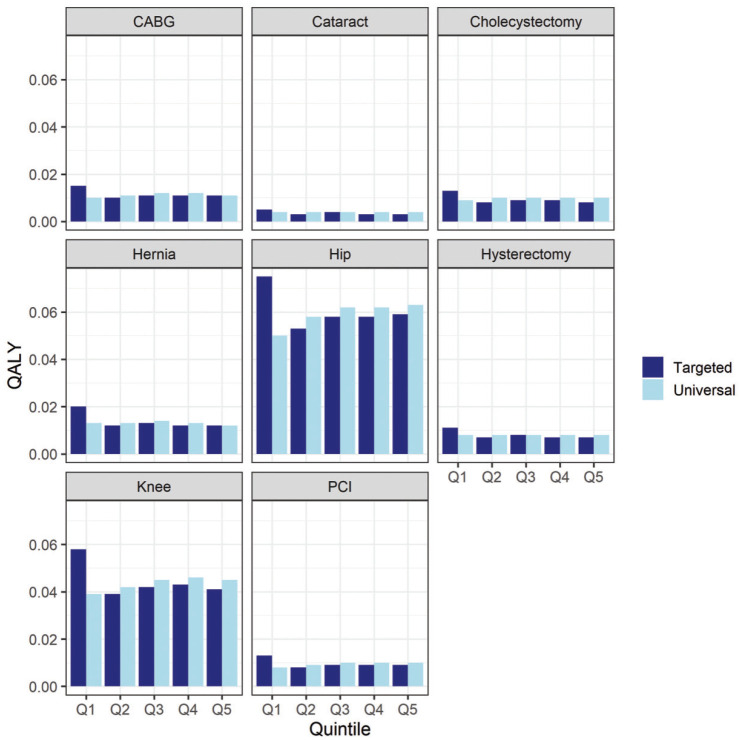
Procedure and index of multiple deprivation quintile group increase in mean individual lifetime quality-adjusted life-years for patients, comparing the universal and targeted policy.

The reduction in waiting time resulted in an increase in QALYs for all individuals across both policy scenarios (compared with baseline; Figure 2), as expected. The targeted policy, compared with the universal policy, resulted in a larger increase in mean individual health for IMD quintile 1 (most deprived), such that an individual in that group now realizes the largest health gain across all groups. This relatively larger health gain to quintile 1, comparing the targeted policy with the universal policy, was offset by a reduction in the health gain to the other 4 quintiles.

The population-level health gains were bigger under the universal policy for 6 of the procedures but lower for CABG and hernia (Table 5). Aggregating across all 8 procedures, the universal policy resulted in slightly higher health gains than the target policy did, but the difference was small. For the targeted policy, the increase in QALYs in Q1 was not sufficient to outweigh the reduction in QALY gains in Q2 to Q5 from the wait-time redistribution. This was primarily driven by people living in more deprived areas benefitting less due to worse mortality and health-related quality of life.

**Table 5:** Average Individual Health Gain (across the Full Population, Not Only Patients) and Change in the Slope Index of Inequality by Procedure

	Average Individual Level Health Gain (QALYs)		Average Individual-Level Change in the Slope Index of Inequality	
	Universal	Targeted	Universal	Targeted
CABG	0.14e-5	0.14e-5	0.01e-5	0.00e-5
Cataract	2.30e-5	2.02e-5	0.26e-5	0.01e-5
Cholecystectomy	2.56e-5	2.44e-5	0.09e-5	−0.19e-5
Hernia	1.50e-5	1.55e-5	0.16e-5	0.04e-5
Hip replacement	7.73e-5	7.62e-5	1.83e-5	1.32e-5
Hysterectomy	0.57e-5	0.56e-5	0.02e-5	−0.03e-5
Knee replacement	6.17e-5	6.12e-5	1.12e-5	0.62e-5
PCI	0.36e-5	0.36e-5	0.06e-5	0.02e-5
TOTAL	21.32e-5	20.81e-5	0.44e-5	0.22e-5

CABG, coronary artery bypass graft; PCI, percutaneous coronary intervention; QALY, quality-adjusted life-year.

The SII increased for all procedures for the universal policy, meaning health inequality increased (Table 5). The targeted policy resulted in a smaller increase in the SII than the universal policy did, whereas for hysterectomy and cholecystectomy, the SII change was negative, implying a reduction in health inequality. Aggregating all the health impacts across the procedures and calculating the SII indicated that both policies increase inequality, but the targeted policy increased inequality less than the universal policy did (Table 5). This overall impact occurred despite the individual-level comparisons for the targeted policy (Figure 2) favoring the most deprived group. Due to the uneven distribution of patients across the quintiles (Table 1), once the individual differences were multiplied by patient population size, we observed an increase in inequality even for the targeted policy. The extent to which this was driven by the relative prevalence of procedures across IMD quintile groups was explored in the scenario analysis. To put these results in context, the population-level SII in England at baseline was 114 million QALYs (i.e., the least deprived quintile had 114 million QALYs more than the most deprived quintile did). At an individual level, health inequality in the United Kingdom suggests that a typical person from the least deprived quintile enjoys 13.84 more QALYs than a typical person from the most deprived quintile does. Altering waiting times for elective procedures produces numerically small impacts at the individual level, in addition to which the elective procedures we considered affect a small proportion of people within each IMD quintile. However, they are still important to consider in prioritization decisions and at a population level, as these small individual effects add up to 12,044 QALYs for the universal policy and 11,752 QALYs for the pro-poor policy.

For ease of comparison, between policies, the results are reported times 10^−5^.

**Figure 3:**
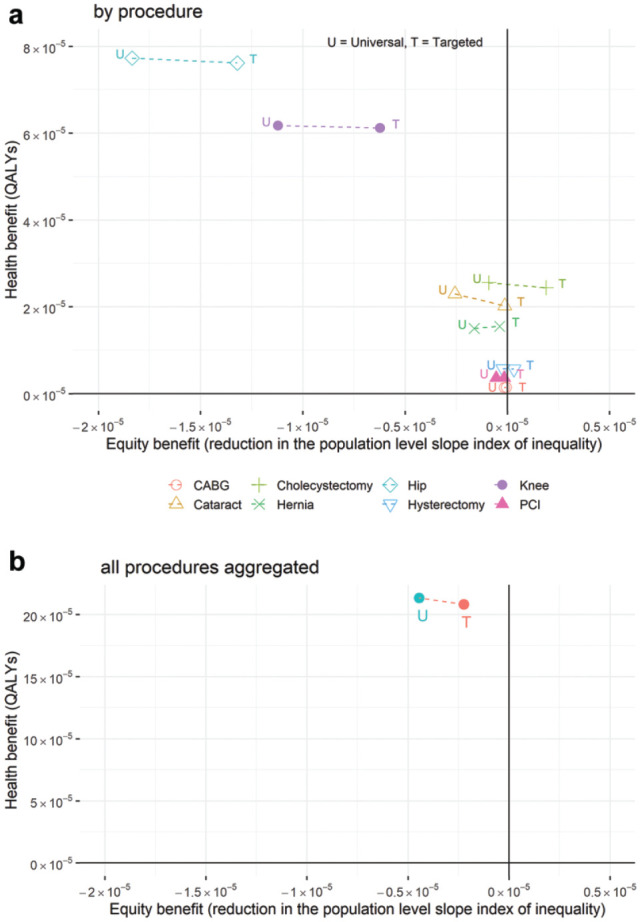
Health gain versus inequality impact for universal versus targeted wait-time reduction policies: (a) by procedure and (b) all procedures aggregated.

The relationship between health benefit and health inequality impact can be illustrated on an equity impact plane (Figure 3). The *y*-axis represents the impact on population health, and the *x*-axis represents a reduction in the SII. We see that the wait-time reduction policies improved population health across all procedures, with hip and knee replacements having the greatest impact on health (Figure 3a). It is clear that for almost all procedures, there was a negative impact on inequality (with a negative reduction in the SII, i.e., an increase in inequality). The targeted policy shifted the inequality impact to the right, indicating a less negative impact on inequality or, in the case of cholecystectomy and hysterectomy, a positive impact on health inequality.

The aggregated impact across all 8 procedures was plotted on a separate equity impact plane (Figure 3b). This allows us to see clearly that there was very little overall health loss for the targeted policy compared with the universal policy, and it had less of a negative impact on health inequalities.

In the sensitivity analysis, equalizing the patient population across quintiles gave a very similar health gain between the universal and targeted policies, whereas the impact on health inequality was more marked, moving from an increase in inequality for the uniform policy (although this is close to zero) to reducing inequality for the targeted policy (Appendix Table 2 and Figure 1). This implies that the inequality impact of changing waiting times was driven largely, though not fully, by the relative size of the patient populations requiring a procedure by IMD quintile group.

## Discussion

We have estimated that a universal reduction in waiting time for 8 elective procedures in the NHS in England may improve health but increase population-level health inequality, measured in QALYs. This is true for each of the 8 procedures and on aggregate. The increase in inequality is due to a combination of factors related to the differential mortality and morbidity effects by quintile group and the relatively larger share of less deprived groups among the patient population. A targeted policy, in which the most deprived quintile receives a greater reduction in wait time than the other 4 quintiles do, would achieve almost the same overall health gain but with a lesser increase in population-level health inequality in aggregate. Looking at the impact of the targeted policy on each of the 8 procedures separately, it always increases health inequality by less than the universal policy and in 2 cases reduces health inequality.

Given the larger share of elective procedures among those living in less deprived areas, we might expect that increasing provision in an unguided manner would provide more benefits to these less deprived groups. If the government is committed to reallocating resources within elective procedures, then a policy targeting those living in more deprived areas would lessen the health inequality increase or potentially even decrease health inequality depending on the strength of the policy.

There may be feasibility concerns surrounding prioritizing specific groups on an elective waiting list. It might be a challenging policy to implement given that most NHS waiting lists are currently prioritized according to severity^21^; therefore, incorporating a concern for deprivation may require a systematic shift in practice. In addition to feasibility, it may not be politically palatable to put people from the most deprived IMD quintile to the front of the queue. COVID-19 highlighted problems with health inequalities, which put reducing this gap back to the top of the agenda. Having a targeted waiting times policy in exceptional times, such as following a pandemic, may be more acceptable than an ongoing policy in “regular” times. Finally, it may be that reducing waiting by 1 wk for the most deprived is not perfectly exchangeable with reducing waiting by 1 wk for less deprived patients. In this case, there would be a lower reduction in overall wait across all patients, and therefore, a lower level of overall health would be achieved. However, the resources required to provide the initial procedure should be similar across IMD quintile group, and evidence is mixed regarding postprocedure resource use, for example, length of stay,^22,23^ suggesting that within-hospital resource use may be relatively comparable across IMD quintile group.

There are a number of limitations linked to the modeling methods and the data, which are listed in detail in previous publications.^1,14^ The model simulates outcomes for a snapshot in time. There is no adjustment made to the proportion of the populations in each quintile that would result from targeting the most deprived quintile. In addition, our baseline is drawn from 2019–2020 data and therefore underestimates current post-COVID wait times, although the lessons still hold. The sensitivity analysis we have included demonstrates how much of the inequality impact relates to baseline prevalence by IMD quintile group, but additional sensitivity analysis may also have been valuable. In particular, the research would benefit from fully probabilistic results allowing for parameter uncertainty to be illustrated, although we expect this would be far outweighed by the distribution of prevalence at baseline. Structural uncertainty in the model is also an area for further research; for example, we have been able to model only a limited number of reasons for exit, but there may be additional ones we have not captured. Other limitations include having access to more data for some procedures (such as hip and knee replacements) than others, having minimal data on patients on the waiting list who never receive the procedure (due to death, exit to private or acute care), inclusion of privately funded procedures and valuing their outcomes equally, and waiting including only the inpatient wait, which misses a substantial period of waiting that may be patterned by deprivation. Given the data available to us, we have used an area-level measure of deprivation, but ideally, we would have deprivation measured at an individual patient level.

An additional key limitation is the omission of the costs of changing waiting times. We can reasonably assume that the resources needed to reduce the waiting time for a CABG are not the same as that of a cataract. Currently our scenarios suggest exchanging waiting between patients only within a procedure, not between procedures. However, a broader analysis could consider between-procedure substitution. In addition, health care resource use costs from acute care or ongoing symptom management could vary substantially. We would also need to include the opportunity costs of the policy and on whom they fall.^24^

In relation to our use of the model to simulate outcomes for 2 hypothetical policy scenarios, we identify a few more limitations. First, our model uses mean wait, whereas the current 18-wk policy is focused on making sure 92% of patients receive their procedure in that time (i.e., it is concerned with the tail of the distribution). In using mean wait, we assume that the distribution of wait does not change and a shift in the mean shifts the tail.

The policy document^10^ that motivated our scenarios includes ethnicity as a focus alongside deprivation, and we have focused only on deprivation. This is due to high levels of missing data on ethnicity in HES, which we found to be getting worse over time. We know there is a significant intersection between ethnicity and deprivation, and so we expect focusing on the most deprived quintile will include higher numbers of the global majority population; nevertheless, there is important intersectionality that is missing from our analysis.

## Conclusion

We have estimated the impact of a universal versus targeted policy for reducing waiting for elective procedures in England on health maximization and health inequalities measured in QALYs. The NHS policy to focus on the most deprived quintile in the recovery of elective procedures may have lessened the negative impact on health inequalities while largely maintaining the health gain achievable from a policy in which all groups are treated equally.

## Supplemental Material

sj-docx-1-mpp-10.1177_23814683241310146 – Supplemental material for Prioritizing Patients from the Most Deprived Areas on Elective Waiting Lists in the NHS in England: Estimating the Health and Health Inequality Impact Supplemental material, sj-docx-1-mpp-10.1177_23814683241310146 for Prioritizing Patients from the Most Deprived Areas on Elective Waiting Lists in the NHS in England: Estimating the Health and Health Inequality Impact by Naomi Kate Gibbs, Susan Griffin, Nils Gutacker, Adrián Villaseñor and Simon Walker in MDM Policy & Practice
