# Supplementary material for: Prioritizing Patients from the Most Deprived Areas on Elective Waiting Lists in the NHS in England: Estimating the Health and Health Inequality Impact
Source: MDM Policy Pract. 2025 Jan 21;10(1):23814683241310146. doi: 10.1177/23814683241310146 (PMC11748141; doi:10.1177/23814683241310146)

# Appendix

Sensitivity analysis

In our sensitivity analysis we aggregate the total estimated population for each procedure and redistribute so that the population is equal in each quintile. We reproduce Table 1 with the new quintile populations.

Appendix table 1: Estimated numbers entering the waiting list (and percentage share), with populations equal across quintiles

|  | Q1 (poorest) | Q2 | Q3 | Q4 | Q5 |
| --- | --- | --- | --- | --- | --- |
| CABG | 1417 (20%) | 1417 (20%) | 1417 (20%) | 1417 (20%) | 1417 (20%) |
| Cataract | 64899 (20%) | 64899 (20%) | 64899 (20%) | 64899 (20%) | 64899 (20%) |
| Cholecystectomy | 29432 (20%) | 29432 (20%) | 29432 (20%) | 29432 (20%) | 29432 (20%) |
| Hernia | 13091 (20%) | 13091 (20%) | 13091 (20%) | 13091 (20%) | 13091 (20%) |
| Hip replacement | 14485 (20%) | 14485 (20%) | 14485 (20%) | 14485 (20%) | 14485 (20%) |
| Hysterectomy | 8008 (20%) | 8008 (20%) | 8008 (20%) | 8008 (20%) | 8008 (20%) |
| Knee replacement | 15862 (20%) | 15862 (20%) | 15862 (20%) | 15862 (20%) | 15862 (20%) |
| PCI | 4293 (20%) | 4293 (20%) | 4293 (20%) | 4293 (20%) | 4293 (20%) |

The results when setting the quintile specific populations show, as expected, that the targeted policy has a bigger impact on reducing health inequality outcomes, with a reduction in inequality for each of procedure (Appendix table 2).

Appendix table 2: Population health gain and change in the Slope Index of Inequality by procedure, with populations equal across quintiles

|  | Population level health gain (QALYs) | | Change in the slope index of inequality (SII) | |
| --- | --- | --- | --- | --- |
|  | Universal | Targeted | Universal | Targeted |
| CABG | 0.14e-5 | 0.15e-5 | 0.00e-5 | -0.01e-5 |
| Cataract | 2.30e-5 | 2.07e-5 | 0.00e-5 | -0.23e-5 |
| Cholecystectomy | 2.55e-5 | 2.45e-5 | 0.05e-5 | -0.23e-5 |
| Hernia | 1.51e-5 | 1.60e-5 | -0.02e-5 | -0.19e-5 |
| Hip replacement | 7.56e-5 | 7.77e-5 | 0.38e-5 | -0.35e-5 |
| Hysterectomy | 0.57e-5 | 0.57e-5 | 0.00e-5 | -0.06e-5 |
| Knee replacement | 6.09e-5 | 6.26e-5 | 0.22e-5 | -0.42e-5 |
| PCI | 0.36e-5 | 0.36e-5 | 0.02e-5 | -0.03e-5 |
| TOTAL | 21.08e-5 | 21.22e-5 | 0.08e-5 | -0.18e-5 |

The procedures with the universal policy all increase the slope index of inequality although this is very close to zero for all procedures except knee and hip replacements. Following the targeted policy they all reduce the slope index of inequality. The biggest change is for hip and knee replacements (Appendix figure 1).

Appendix figure 1: Total health gain versus inequality impact for universal versus targeted wait time reduction policies, with populations equal across quintiles


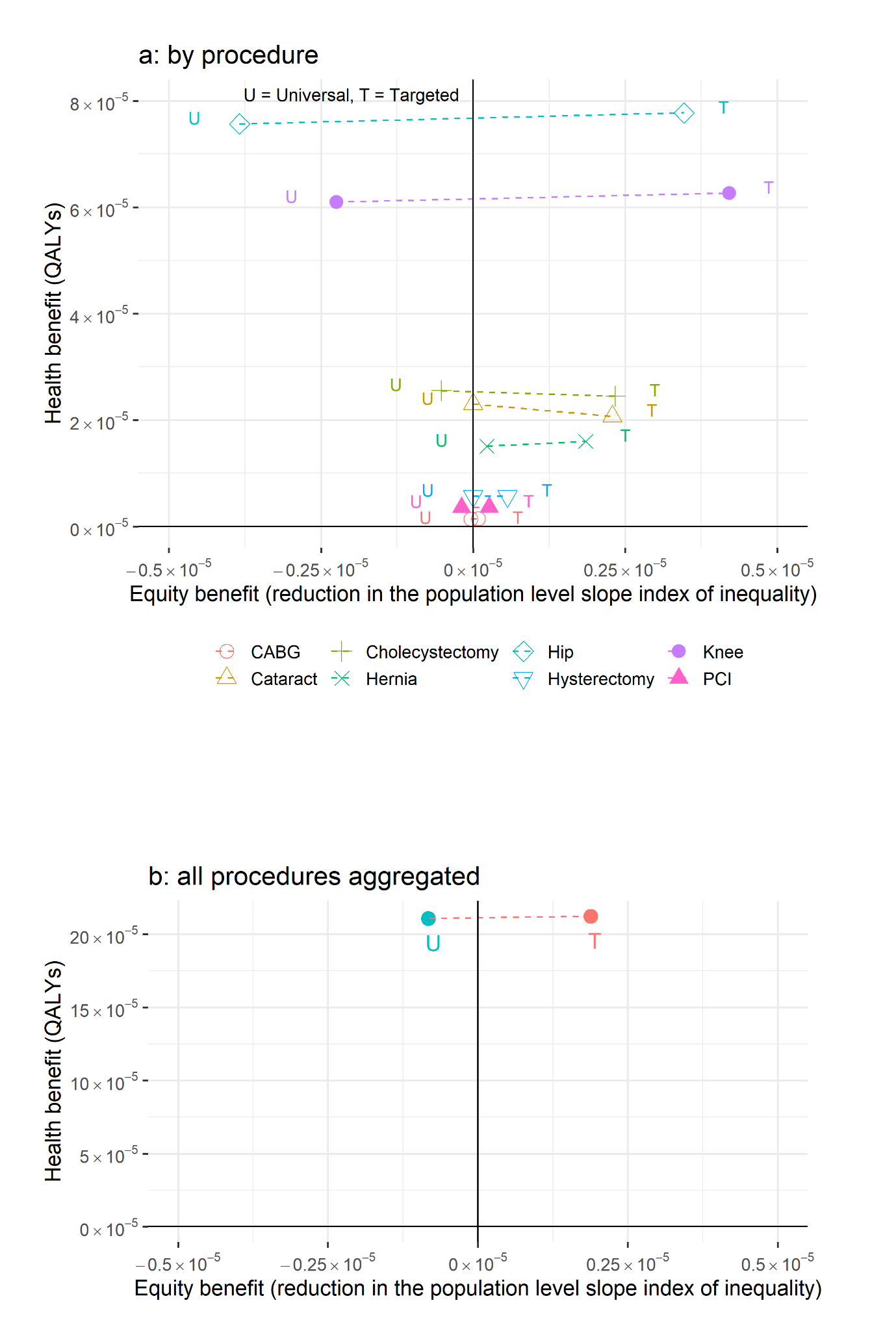

Supplement: sj-docx-1-mpp-10.1177_23814683241310146 – Supplemental material for Prioritizing Patients from the Most Deprived Areas on Elective Waiting Lists in the NHS in England: Estimating the Health and Health Inequality Impact [file sj-docx-1-mpp-10.1177_23814683241310146.docx]
